# Supplementary material for: CRISPR/Cas9-Mediated Knockout of the White Gene in Agasicles hygrophila
Source: Int J Mol Sci. 2025 May 10;26(10):4586. doi: 10.3390/ijms26104586 (PMC12110819; doi:10.3390/ijms26104586)
Supplement: Supplementary file 1 [file ijms-26-04586-s001.zip › ijms-3551745-supplementary.pdf]

**Supplementary Materials**

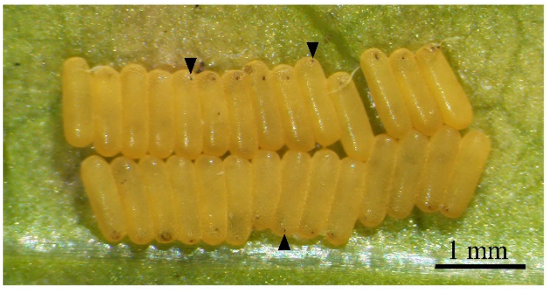


**Figure S1**. Microinjected beetle eggs. The black arrow shows the needle hole after microinjection

**Table S1.** Primer sequences used in this study.

| | Sequence 5'-3' | Destination |
|---|---|---|
| AhW-F | AACACATAAAATTTTTGTACGATGATATCAG | AhW clone |
| AhW-R | CATATAAAATAATATACTATGGAAAGTACAGG | |
| gRNA-F1 | AAGCTTC<u>TAATACGACTCACTATA</u>GG**ATTTAACTATAACTGGTAGG***GTTTTAGAGCTAGAAATAGCA* | gRNA synthesis |
| gRNA-F2 | AAGCTTC<u>TAATACGACTCACTATA</u>GG**GGACGAAGGTGGTATCTCAG***GTTTTAGAGCTAGAAATAGCA* | |
| gRNA-R | *ATAACGGACTAGCCTTATTTTAACTTGCTATTTCTAGCTCTAAAAC* | |
| AhW-qF | TGGACAAGAGATGAATCAA | qPCR |
| AhW-qR | CTCACGAAGGAACACTAA | |
| RPS18-qF | ACAAAATCCCCGACTGGTTC | |
| RPS18-qR | ATGGGCACGGATCTTCTTCA | |
| AhW-mutF | AATATGGTAGCGAAAGTAGAGCGTCTAAAT | Mutant identification |
| AhW-mutR | TATTAACGACACCGCTTGTCTACACGATTC | |

The T7 promoter is underlined, gRNA is in bold, and the sgRNA scaffold is in italics.

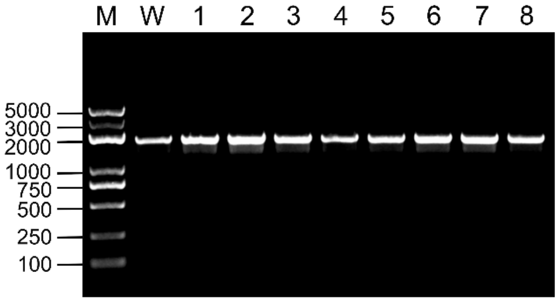


**Figure S2.** Electrophoresis of *AhW* editing site sequences of G1. The genomic DNA of wild-type and G1 white-eye male beetle was extracted, and then the sequences of the *AhW* editing sites were amplified and electrophoresized. M, DNA marker; lane W wild-type, lane 1-8, *AhW* KO1-KO8 of G1 white-eyed mutant.

**Table S2.** The accession numbers of the protein sequences used in the phylogenetic tree.

| Gene name | Species | Accession number |
|---|---|---|
| White | *Agasicles hygrophila* | OR123872 |
| | *Dendroctonus ponderosae* | XP_019773198.2 |
| | *Tribolium castaneum* | NP_001034521.1 |
| | *Asbolus verrucosus* | RZB77475.1 |
| | *Dorcus rectus* | BDX53024.1 |
| | *Onthophagus taurus* | XP_022907669.1 |
| | *Rhyzopertha dominica* | KAI7815062.1 |
| | *Drosophila melanogaster* | NP_476787.1 |
| | *Bactrocera dorsalis* | QPB75811.1 |
| | *Anopheles gambiae* | Q27256.1 |
| | *Bombyx mori* | BAH03523.1 |
| | *Helicoverpa armigera* | XP_049695076.1 |
| | *Plodia interpunctella* | XP_053625431.1 |
| | *Papilio xuthus* | KPJ00092.1 |
| Scarlet | *Drosophila melanogaster* | NP_524108.1 |
| | *Anopheles gambiae* | XP_310585.5 |
| | *Tribolium castaneum* | NP_001306193.1 |
| | *Dendroctonus ponderosae* | XP_019773228.2 |
| | *Bombyx mori* | NP_001243922.1 |
| | *Helicoverpa armigera* | XP_063892436.1 |

| | | |
|---|---|---|
| Brown | *Drosophila melanogaster* | NP_523824.1 |
| | *Anopheles gambiae* | XP_061502693.1 |
| | *Tribolium castaneum* | AJD07061.1 |
| | *Bombyx mori* | BAN66702.1 |
